# Porcine sclera as a model of human sclera for in vitro transport experiments: histology, SEM, and comparative permeability

**Published:** 2009-02-06

**Authors:** S. Nicoli, G. Ferrari, M. Quarta, C. Macaluso, P. Govoni, D. Dallatana, P. Santi

**Affiliations:** 1 Department of Pharmacy, University of Parma, Italy; 2 G.B. Bietti Eye Foundation, IRCCS, Rome, Italy; 3 Department of Ophthalmology, University of Parma, Italy; 4 Department of Experimental Medicine, Section of Histology, University of Parma, Italy; 5 Department of Human Anatomy, University of Parma, Italy

## Abstract

**Purpose:**

To evaluate porcine sclera as a model of human sclera for in vitro studies of transscleral drug delivery of both low and high molecular weight compounds.

**Methods:**

Human and porcine scleras were characterized for thickness and water content. The tissue surface was examined by scanning electron microscopy (SEM), and the histology was studied with hematoxylin-eosin staining. Comparative permeation experiments were performed using three model molecules, acetaminophen as the model compound for small molecules; a linear dextran with a molecular weight of 120 kDa as the model compound for high molecular weight drugs; and insulin, which was chosen as the model protein. Permeation parameters such as flux, lag time, and permeability coefficient were determined and compared.

**Results:**

Human and porcine scleras have a similar histology and collagen bundle organization. The water content is approx 70% for both tissues while a statistically significant difference was found for the thickness, porcine sclera being approximately twofold thicker than human sclera. Differences in thickness produced differences in the permeability coefficient. In fact, human sclera was found to be two to threefold more permeable toward the three molecules studied than porcine sclera.

**Conclusions:**

The results obtained in the present paper prove that porcine sclera can be considered a good model for human sclera for in vitro permeation experiments of both low and high molecular weight compounds. In fact, if the different tissue thickness is taken into account, comparable permeability was demonstrated. This suggests a possible use of this model in the evaluation of the transscleral permeation of new biotech compounds, which currently represent the most innovative and efficient therapeutic options for the treatment of ocular diseases.

## Introduction

Transscleral administration is considered a possible non-invasive alternative to injection to target the posterior segment of the eye for the treatment of chorioretinal diseases. To test the feasibility of this administration route, the pharmacokinetic and the complex ocular structure impose several steps of in vitro and in vivo experimentation. The first preliminary step in the development of transscleral delivery is represented by in vitro permeation studies through isolated sclera. The reference tissue for these studies is human sclera, although its limited availability often imposes the use of animal models [1,2]. Different models are currently used. Rabbit is the most commonly reported model, but also cow [3] and pig are present in the literature [2]. Porcine sclera has been thoroughly characterized in terms of thickness by Olsen [4] and was found to be similar to human sclera. However, few data are available on its permeability [2,5,6]. More specifically, to our knowledge, no literature data are present concerning the permeability toward high molecular weight compounds, which currently represent the most innovative and promising drugs for the treatment of posterior segment eye diseases [7].

The aim of this work was to characterize and compare human and porcine scleras to verify adequacy, reliability, and predictivity of porcine sclera for in vitro permeation experiments. The characterization included histology, scanning electronic microscopy (SEM) of the outer region of the sclera, and measurement of water content. Moreover, the permeability of three different model molecules through the two tissues was studied. The molecules chosen were acetaminophen as the model compound for small molecules, a linear dextran with a molecular weight of 120 kDa as the model compound for high molecular weight drugs, and insulin, which was chosen as the model protein and also for its potential use in the treatment of diabetic retinopathy [8,9].

## Methods

### Materials

Insulin from bovine pancreas, fluorescein isothiocyanate (FITC)-dextran (FD-150; effective MW: 120 kDa) acetaminophen, HEPES (4-[2-hydroxyethyl]-1-piperazineethanesulfonic acid), lysine, and EDTA were purchased from Sigma (St. Louis, MO). For HPLC analysis, acetonitrile (HPLC grade) and distilled water were used. All other chemicals used were of analytical grade.

### Tissue preparation

Porcine globes were obtained from pigs (Large White, Landrance, Duroc; 10–11 months) that ranged in weight 145–190 kg and came from a local slaughterhouse. Porcine eyes were either used within 24 h of explantation or frozen at −80 °C until use. After thawing, the adherent muscle tissue was removed from the eye bulb, and the anterior segment of the eye was circumferentially cut behind the limbus. The eye was then cut into two halves, the vitreous was removed, and the anterior sclera was used for permeation experiments after the removal of the underling tissues using a cotton swab. The frozen tissues were used within three months of explantation.

Human corneal-scleral rims, which were discarded following harvesting of the corneal button (Regional Cornea Bank, Bologna, Italy), were frozen in liquid nitrogen and used within 15 days of explantation.

### Tissue characterization

Human and porcine scleras were characterized in terms of thickness and water content. The thickness was measured with a digital caliper (resolution 0.001 mm; Absolute Digimatic 547–401; Mitutoyo, Milan, Italy) at the limbus, equator, and posterior pole of the porcine eye bulb, and the average value was calculated. In the case of human sclera, a single measurement for each sample was performed since the specimens have a limited size. Thickness measurements were performed before and after freezing.

For the calculation of water content of the tissue, the sclera was weighed and then dried in a dessiccator in the presence of P_2_O_5_ to constant weight. The water content (%) is the mass of water per unit mass of the moist specimen:

$$\text{water content }\%=\frac{{\text{w}}_{\text{i}}{\text{-w}}_{\text{f}}}{{\text{w}}_{\text{i}}}\text{ x 100}$$

where w_i_ and w_f_ are the initial and final weight, respectively.

### Optical microscopy

Pieces of human and porcine sclera (fresh or previously frozen) were fixed in 10% formaldehyde and then embedded in paraffin before sectioning (6 μm thick slices) using a microtome. All sections were then stained using Harris hematoxylin/eosin.

Images were taken using an optical microscope Nikon Eclipse 80i (Nikon Instruments, Calenzano, Italy) equipped with a camera, Nikon Digital Sight DS-2Mv, connected to the control software, NIS-Elements F (Nikon Instruments).

### Scanning electron microscopy

Samples of scleral tissue, which were previously frozen in liquid nitrogen, were thawed and fixed in 10% formaldehyde then dehydrated in alcohol, then in absolute acetone, and treated with a critical point dryer in liquid CO_2_. The specimens were Au-metallized with a sputtering device. The observation was performed with a scanning electron microscope (Philips, Scansion Electron Microscope, model 501; Philips, Hamburg, Germany).

### Permeation experiments

Permeation experiments were performed in Franz-type diffusion cells with an area of 0.2 cm^2^ for human sclera and 0.6 cm^2^ for porcine sclera. Preliminary control experiments demonstrated that the size of the cell area did not influence drug permeation.

To reduce edge damage, a thin layer of silicone lubricant was applied to the glass surface, and the minimum force necessary to keep the cell sealed was applied.

The donor compartment contained alternatively 4.9 mg/ml acetaminophen, 1 mg/ml 120 kDa FITC-Dextran (FD-150), or 1 mg/ml insulin dissolved in 25 mM HEPES buffer at pH 7.4. To improve insulin solubility in this vehicle, 0.4 mM disodium EDTA and 0.3 mM lysine were added [10].

The receptor compartment contained 4 ml of 25 mM HEPES buffer (pH 7.4) added with 0.9% NaCl, kept at 37 °C, and magnetically stirred. In the case of insulin, 0.3% w/v bovine serum albumin (BSA) was added to the receptor phase to increase the solubility of the protein. At predetermined time intervals, the receptor solution was sampled for the determination of drug permeated.

The transscleral flux, i.e., the amount of drug that crosses 1 cm^2^ of sclera in 1 h (J, µg/cm^2^h) was calculated as the slope of the regression line at the steady-state while the lag time was the intercept of the regression line on the x-axis. The permeability coefficient (P, cm/s) was calculated as J/C_v_ where C_v_ represents the concentration of the donor solution.

Experiments performed with human sclera were replicated three to four times while those performed on porcine sclera were replicated five to seven times. All the experiments were performed using previously frozen tissues, and acetaminophen permeability was tested also through fresh pig sclera.

### Analytical methods

Acetaminophen and insulin were analyzed by HPLC with a Perkin-Elmer instrument (Norwalk, CT), which is made of an isocratic pump LC250 an UV detector LC290, and the Perkin Elmer Turbochrom Workstation software.

Acetaminophen was analyzed using a C18 Novapak^®^ column (150×3.9 mm; Waters, Milford, MA) and a mobile phase composed of 92% (v/v) 10 mM sodium acetate pH 4 and 8% (v/v) acetonitrile, which was pumped at 1 ml/min. The detector was set at 254 nm.

Insulin was analyzed using a C18 Symmetry300^®^ column (250×4.6 mm; Waters) at 40 °C and a mobile phase composed of 73% (v/v) aqueous phase and 27% (v/v) acetonitrile, which was pumped at 1 ml/min. The aqueous phase contained anhydrous sodium sulfate 28.4 g/l and phosphoric acid 2.7 ml/l and was adjusted at pH 2.3 with ethanolamine. The detector was set at 214 nm.

FD-150 was analyzed with a fluorescence detector (Series 200a Perkin Elmer). The excitation and emission λ were 490 and 520 nm, respectively.

### Affinity of insulin for the scleral tissue

Affinity of insulin for scleral tissue was estimated by measuring its partition coefficient between human or porcine sclera and an aqueous solution at pH 7.4. Insulin was dissolved in the aqueous vehicle that was used as donor for the permeation experiments at a concentration of about 100 µg/ml. This solution (0.4 ml) was added to a previously weighed amount of human or porcine sclera (50–100 mg) in a 2.0 ml vial. The insulin solution and the scleral tissues were incubated at room temperature for 3 h. The solution was then filtered (regenerated cellulose, 0.45 µm pore size) and analyzed by HPLC for the determination of the final insulin concentration in the water phase [W_f_]. The insulin concentration [S] in the sclera after the incubation period was calculated as:

$$[S]=\frac{[{\text{W}}_{\text{i}}]{\text{ x V}}_{\text{W}}\text{ - }[{\text{W}}_{\text{f}}]{\text{ x V}}_{\text{W}}}{{\text{V}}_{\text{S}}}$$

where [W_i_] is the initial concentration of insulin in the aqueous phase, [W_f_] is the concentration of insulin in the aqueous phase after the incubation period, V_W_ is the volume of the aqueous phase (0.4 ml), and V_S_ is the volume of the sclera, which was estimated by its weight and by considering the scleral density to be equal to 1 g/ml.

The partition coefficient (K_s/w_) was then calculated as:

$${\text{K}}_{\text{s}/\text{w}}=\frac{\left[S\right]}{\left[{\text{W}}_{\text{f}}\right]}\text{ x }\frac{{\text{V}}_{\text{W}}}{{\text{V}}_{\text{S}}}$$

Each experiment was replicated at least six times.

### Statistical analysis

The significance of the differences between values was assessed using one-way ANOVA followed by Bonferroni test (Kaleidagraph 4.01 software [Synergy Software, Reading, PA] on a Macintosh iBook G4 [Apple Computers, Cupertino, CA]). Differences were considered statistically significant when p<0.05.

## Results

### Tissue characterization

The mean thicknesses of porcine and human scleras (measured after thawing of the frozen tissue) were 1.25±0.25 mm and 0.59±0.08 mm, respectively. Some specimens were also measured before freezing, and the values obtained were not statistically different from the frozen tissue.

The water content in the two species is comparable, 71.6%±0.63% for human sclera and 69.5%±1.18% for porcine sclera.

### Optical microscopy

**Figure 1:**
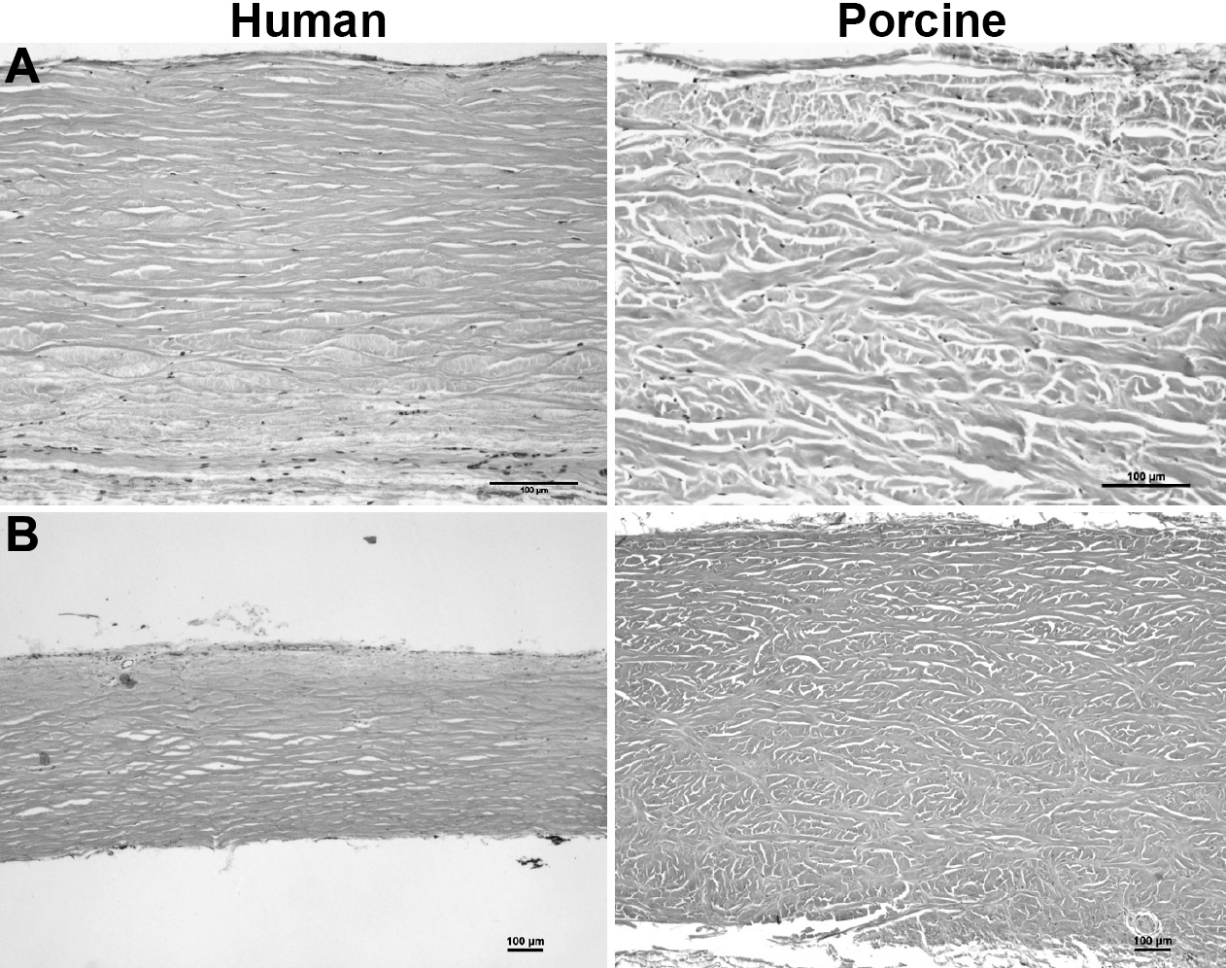
Histological microscopic sections of human and porcine sclera stained with hematoxylin-eosin. Empty lacunae between fibers are an artifact due to tissue preparation.  Original magnification was 10X in panel **A** and 4X in panel **B**. In both human and porcine scleras, scattered small fibrocyte nuclei are dispersed between the bundles of interwoven collagen fibers. In porcine sclera, thicker and more disorganized collagen bundles are visible. From the 4X magnification (**B**) it is possible to appreciate differences in thickness between human and porcine sclera.

Histology confirmed the similarity between human and porcine scleras, although globally thicker and more disorganized collagen bundles were found in porcine sclera (Figure 1). Empty lacunae between fibers in both human and porcine sclera are an artifact due to tissue preparation. Scattered, small fibrocyte nuclei were dispersed between the bundles of interwoven collagen fibers. Human specimens show thick and interlaced bundles between the episclera and the choroid. Porcine and human scleral specimens were analyzed before and after freezing, and no differences were found in the histology of the tissues (data not shown).

### Scanning electron microscopy

**Figure 2:**
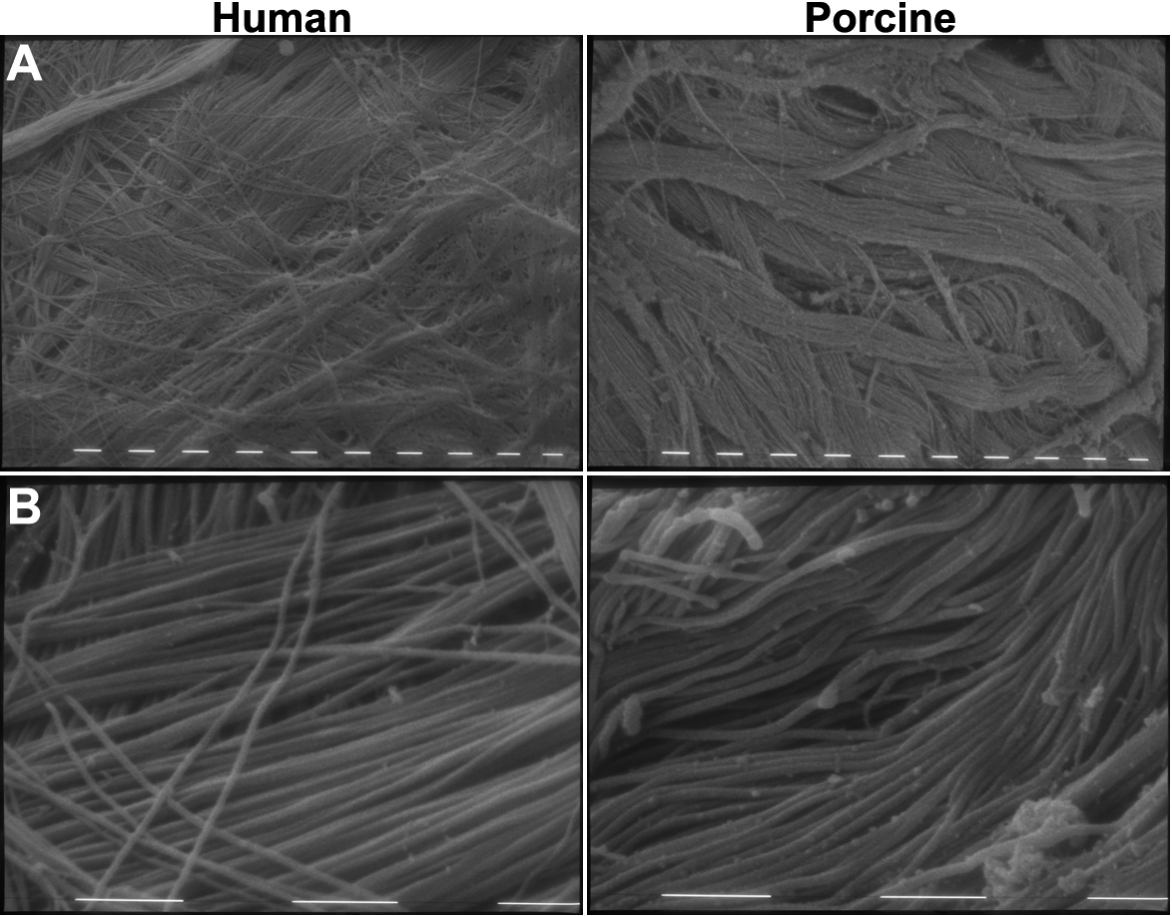
SEM image of the outer region of human and porcine sclera.  Original magnification was 5,000X in panel **A** and 20,000X in panel **B**. Scale bar=10 µm in both cases. From the pictures at lower magnification (**A**) it is possible to observe branching and anastomosis of the collagen bundles to form dense connective tissue. The bundles were of varying thickness and width and often intertwined with each other. Porcine bundles looked thicker at lower magnification, but still showed a similar arrangement. Moreover, higher magnification (**B**) did not show any difference between human and porcine sclera in the diameter of the single collagen fibers

Scanning electron microscopy (SEM) is a very useful technique to study the three dimensional (3D) arrangement of the collagen lamellae of the outer sclera. From the pictures (Figure 2), it is possible to observe branching and anastomosis of the collagen bundles, which form dense connective tissue. The bundles varied in thickness and width and often intertwined with each other. Porcine bundles looked thicker at lower magnification but still showed a similar arrangement.

### Permeation experiments

To compare the two tissues in terms of permeability, permeation experiments with Franz-type diffusion cells were performed across human and porcine scleras using three model permeants with different characteristics: acetaminophen, insulin, and FD-150. It is worth mentioning that with these diffusion cells, it is not possible to reproduce the natural intraocular pressure (IOP) that, to some extent, can influence the permeation of drugs [11].

**Figure 3:**
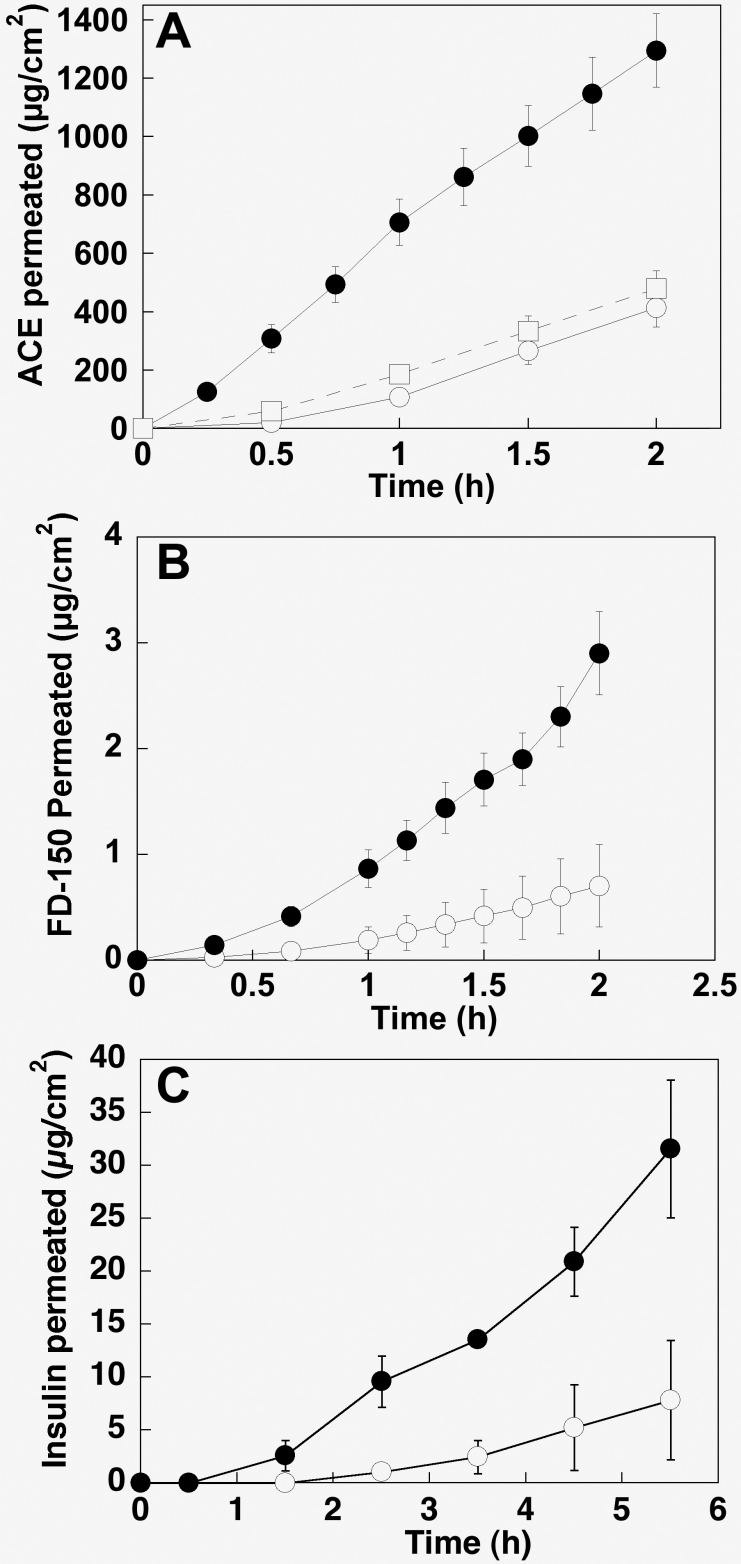
Permeation profiles of acetaminophen, FD-150, and insulin for porcine and human sclera. Profiles for acetaminophen (**A**), FD-150 (**B**), and insulin (**C**) through porcine (○)  and human (●) sclera are shown. Open squares (□) in panel **A** indicate the permeation profile of acetaminophen through fresh porcine sclera. Transscleral fluxes obtained for all three molecules tested were higher for human sclera. In particular, the fluxes through human sclera were 2 times greater than through pig sclera in the case of acetaminophen and insulin, and 3 times greater in the case of FD-150. Permeability of acetaminophen through fresh porcine sclera confirmed that the freezing procedure did not have any effect on tissue permeability. Mean value ±SEM

Figure 3 shows the permeation profiles of the model molecules across human and pig scleras while flux (µg/cm^2^h), permeability coefficient (cm/s), and lag time (min) values are reported in Table 1. Both the data reported in Figure 3 and the values reported in Table 1 show that the transscleral fluxes obtained for all three molecules tested were higher for human sclera even if the differences were not large. In particular, the fluxes (and the permeability coefficients) through human sclera were two times higher than through pig sclera in the case of acetaminophen and insulin and three times higher in the case of FD-150. Together with fluxes and permeability coefficients, a difference between the two species was also found in the lag time. With acetaminophen, no lag time was detectable for human sclera while lag time for pig sclera was 35±2 min. In the case of insulin and FD-150, the lag time of pig sclera were respectively three and two times higher than human sclera.

**Table 1:** Permeation parameters obtained for acetaminophen, FD-150, and insulin through human and porcine scleras from donor solutions at pH 7.4.

**Compound **	**Permeation parameters **	**Human sclera**	**Porcine sclera**	**p value**
**Acetaminophen**	Flux (µg/cm^2^h)	626±55	280±37	<0.001
	P (cm/s) x10^−6^	34.9±3.0	15.6±2.1	0.00047
	DK (cm^2^/s) x10 ^−6^	2.05±0.18	1.95±0.26	0.8043
	Lag-time (min)	-	35±2	-
**FD-150**	Flux (µg/cm^2^h)	1.72 0.23	0.57±0.03	0.0147
	P (cm/s) x10^−6^	0.48±0.067	0.16±0.072	0.0147
	DK (cm^2^/s) x10^−6^	0.028±0.004	0.019±0.009	0.4885
	Lag-time (min)	28±7	57±7	0.0265
**Insulin**	Flux (µg/cm^2^h)	6.93±1.14	3.74±2.10	0.1786
	P (cm/s) x10^−6^	1.93±0.32	0.85±0.48	0.1786
	DK (cm^2^/s) x10^−6^	0.11±0.02	0.11±0.05	0.9321
	Lag-time (min)	71±6	198±23	<0.001

Each value represents the mean±SEM.

Permeability of acetaminophen was also measured through fresh porcine sclera and confirmed that the freezing procedure did not have any effect on tissue permeability (Figure 3A).

### Affinity of insulin for scleral tissue

The permeation profiles of insulin were characterized by a long lag time, indicating a possible interaction between insulin and the scleral tissue. For this reason, the partition coefficient of insulin between the tissue and the donor solution was measured for both human and porcine scleras and was found to be 10.3±1.2 and 7.0±1.1, respectively. The two values are not statistically different between them (p=0.176), indicating a similar interaction of the two tissues with insulin.

## Discussion

The thickness of porcine and human sclera has already been studied by Olsen and coworkers [4,12] that measured the thickness of big, medium, and small size eye bulbs. Eye size and thickness depended upon animal weight that ranged from 2.8 to 81 kg. In the present work, the average thickness of porcine sclera was higher than that reported by Olsen et al. [4,12] due to the consistently higher weight of the animals used (145–190 kg).

The porcine sclera samples used in this work were twofold thicker than the human sclera samples. Despite this difference, it is worth mentioning that the thickness of porcine sclera is closer to human skin than rabbit sclera, which is currently the most used in vitro model. In fact, rabbit sclera has a mean thickness that is approximately one-tenth of that of human sclera [13,14].

The value of water content found for human sclera is consistent with literature reports [15,16]. Concerning SEM imaging and histology, no significant differences were found between porcine and human scleras.

The permeability was evaluated using three model compounds. Acetaminophen is a model molecule for small molecular weight compounds (MW of 151.2 Da) with a molecular radius of approximately 0.36 nm [17] and a logP n-octanol/water of 0.2–0.89 [18]. Insulin has been chosen as a model protein. It has a molecular weight of 5,734 Da, a molecular radius of the monomeric form of 1.13 nm [19] or 2.0 nm [20], and an isoelectric point of about 5.4. FD-150 is a linear FITC-labeled dextran with high molecular weight (120 kDa) and an estimated molecular radius of 8.5 nm [21]. Permeation experiments were performed using previously frozen tissues since it has been demonstrated that even consecutive freeze–thaw treatments to -80 °C did not significantly affect human scleral permeability [22].

The flux of acetaminophen through human sclera was twice that of porcine sclera (626±55 µg/cm^2^h for human and 280±37 µg/cm^2^h for porcine sclera). The permeability coefficient found across human sclera (3.49 [±0.3] x10^-5^ cm/s) is consistent with literature data. Regardless of the lipophilicity, the permeability of low molecular weight drugs through human sclera is typically in the range of 1.0x10^-5^-4.0x10^-5^ cm/s [23].

The permeability coefficient P (cm/s) is also equal to [24]:

$$P=\frac{DK}{H}$$

where D (cm^2^/s) is the diffusion coefficient of the molecule inside the sclera, K is the partition coefficient of the molecule between the sclera and the donor solution, and H is the diffusional path length of the permeant. H can be approximated to the thickness of the membrane because the sclera is a highly porous tissue. Additionally, the two tissues, human and porcine scleras, have the same architecture in terms of porosity and tortuosity. By knowing the thickness of the barrier, the product, DK, can be calculated. As reported in Table 1, the value, DK, is similar for porcine and human scleras. Therefore, it is reasonable that the difference in permeability coefficient observed is simply due to the different thickness of the two tissues.

This result indicates that porcine and human scleras behave in the same way toward the permeation of a small molecule despite a difference in tissue thickness. The similarity of the two tissues in terms of structure, histology, and collagen fiber architecture corroborates this result, which is also supported by in vitro studies on surgically-thinned human sclera that demonstrated the role of the scleral thickness on transscleral transport [22].

When the permeation experiment was performed using a high molecular weight compound (FD-150), which was characterized by a molecular radius of 8.5 nm, the permeability coefficient was 100 times lower than the permeability coefficient of acetaminophen for both human and porcine scleras. This is in agreement with the dependence of the scleral permeability on the permeant molecular radius [25]. The results on the human sclera are also consistent with literature reports. The permeability coefficient obtained here (4.8 [±0.7] x10-7 cm/s) is similar to that found by Cruysberg et al. [11] (approximately 1.0x10^−7^ cm/s), which is incidentally identical to the value of porcine sclera. Also, in the case of FD-150, the value, DK, which underlines the importance of tissue thickness, was similar for both tissues. This means that porcine and human scleras behave similarly with regards to permeability toward big molecules. This is particularly relevant because the last generation of drugs used or proposed for the treatment of the posterior segment eye diseases are represented by high molecular weight compounds such as oligonucleotides, antibodies, and other proteins.

Finally, the permeability of the sclera to insulin was tested. Insulin has been chosen as a model protein, but it has also been recently proposed for its potential use in the local treatment of diabetic retinopathy [8,9]. Once again, the behavior of human and porcine scleras toward insulin was similar. Despite the permeability coefficient being higher for human sclera, the value of DK is almost identical in the two scleras, indicating the same behavior when the different thickness of the two tissues is taken into account. If the results obtained in terms of permeability are consistent with acetaminophen and FD-150 data, the lag time found was long. The permeation experiments had to be continued for 6 h to achieve steady-state conditions (see Figure 3C). This could be due to several reasons. One is linked to the peculiar behavior of insulin that is able to form dimers and hexamers in solution, which results in the molecular radius of the permeant not being 1.13 nm but possibly about 2.5 nm [26]. Another hypothesis is that insulin interacts with specific or non-specific binding sites on the scleral structures. The presence of specific binding sites for insulin has been demonstrated for chick sclera [27] while non-specific binding to the sclera has been reported for negatively charged molecules (fluorescein and carboxyfluorescein) [6,28] as insulin is at pH 7.4 [26].

Regardless of the nature of this interaction, we have estimated that the affinity of insulin for the sclera was the same for porcine and human tissues. The affinity, measured as the partition coefficient between the sclera and the donor solution, was 10.3±1.2 (mean value±standard error of the mean [SEM]) for human sclera and 7.0±1.1 for porcine sclera. These values (not statistically different between them, p=0.176) indicate a similar behavior of the two tissues toward insulin and also confirm that insulin has an effective affinity for the sclera. In fact, even if the absolute value of the partition coefficient is not particularly high, it is much higher than the partition coefficient values reported in the literature, which are typically equal or lower than 1. Examples of neutral molecules of different lipophilicity are mannitol (0.005), budesonide (1.2), celecoxib (1.4) [2], sucrose (0.6), and FITC-labeled dextrans of various MW (0.07–0.39) [29]. There are some exceptions to these typical values such as rhodamine 6G [2], which showed high affinity for the scleral tissue because it was electrically charged at the pH of the experiment and interacted with the tissue.

Detailed studies performed by Olsen et al. [4,12] have shown important similarities between porcine and human eyes in term of intraocular anatomy and scleral thickness. These similarities are absent in other animal models such as rabbit and cow. Porcine sclera is easily obtained in large amounts from slaughterhouses and can efficiently substitute for rabbit sclera, which is now the most used in vitro model. The results obtained in the present paper further prove that porcine sclera can be considered a good model for human sclera for in vitro permeation experiments. Optical microscopy and SEM showed a similar organization of collagen bundles of the sclera even if they looked thicker and more disorganized in the porcine sample in comparison to the human sample. Moreover, permeation experiments demonstrated that the permeability of porcine and human scleras is very similar toward a small molecule (151 Da), a high molecular weight (120 kDa) compound, and a model protein (5.8 kDa) except for differences in tissue thickness. A very similar binding capacity was also demonstrated toward the model protein, suggesting a possible and reliable use of this model in the evaluation of transscleral drug permeation of new biotech compounds, which currently represent the most innovative and efficient therapeutic options.

Further studies are needed to confirm human and porcine similarities with regards to permeability toward high molecular weight proteins.
